# Structure of the Motor Descending Pathways Correlates with the Temporal Kinematics of Hand Movements

**DOI:** 10.3390/biology11101482

**Published:** 2022-10-10

**Authors:** Chiara Begliomini, Francesco Ceccarini, Veronica Pinuccia Dell’Acqua, Sanja Budisavljevic, Umberto Castiello

**Affiliations:** 1 Department of General Psychology, University of Padova, Via Venezia 8, 35131 Padova, Italy; 2 Padua Neuroscience Center, University of Padova, Via Giuseppe Orus 2, 35131 Padova, Italy; 3 Department of Psychology, New York University Abu Dhabi, Abu Dhabi P.O. Box 129188, United Arab Emirates; 4 Cardiff University Brain Research Imaging Centre (CUBRIC), Cardiff University, Cardiff CF10 3AT, UK

**Keywords:** HMOA, grasping, kinematics, projection system, corticospinal tract

## Abstract

**Simple Summary:**

How hand motor behavior relates to the microstructure of the underlying subcortical white matter pathways is yet to be fully understood. Here we consider two well-known examples of our everyday motor repertoire, reaching and reach-to-grasp, by looking at their temporal unfolding and at the microstructure of descending projection pathways, conveying motor information from the motor cortices towards the more ventral regions of the nervous system. We combine three-dimensional kinematics, describing the temporal profile of hand movements, with diffusion imaging tractography, exploring the microstructure of specific segments of the projection pathways (internal capsule, corticospinal and hand motor tracts). The results indicate that the level of anisotropy characterizing these white matter tracts can influence the temporal unfolding of reaching and reach-to-grasp movements.

**Abstract:**

The projection system, a complex organization of ascending and descending white matter pathways, is the principal system for conveying sensory and motor information, connecting frontal and sensorimotor regions with ventral regions of the central nervous system. The corticospinal tract (CST), one of the principal projection pathways, carries distal movement-related information from the cortex to the spinal cord, and whether its microstructure is linked to the kinematics of hand movements is still an open question. The aim of the present study was to explore how microstructure of descending branches of the projection system, namely the hand motor tract (HMT), the corticospinal tract (CST) and its sector within the internal capsule (IC), can relate to the temporal profile of reaching and reach-to-grasp movements. Projection pathways of 31 healthy subjects were virtually dissected by means of diffusion tractography and the kinematics of reaching and reach-to-grasp movements were also analyzed. A positive association between Hindrance Modulated Orientation Anisotropy (HMOA) and kinematics was observed, suggesting that anisotropy of the considered tract can influence the temporal unfolding of motor performance. We highlight, for the first time, that hand kinematics and the visuomotor transformation processes underlying reaching and reach-to-grasp movements relate to the microstructure of specific projection fibers subserving these movements.

## 1. Introduction

Reaching and reach-to-grasp movements are among the most common actions performed in everyday life. In our eyes, they seem apparently simple and automatic. Yet, they are not. The processes underlying these movements rely on complex “sensorimotor transformations” converting sensory information into motor commands [1,2,3,4].

To perform a successful reaching movement, the central nervous system (CNS) maps into motor output information concerning the hand and the object location and it coordinates the activation of shoulder and arm muscles that bring the hand towards the target [5,6,7]. The reach-to-grasp movement involves an additional grasping component, consisting in a progressive opening of the hand (opening phase), followed by a gradual closure of the grip until it matches the object’s size (closing phase). The possibility of successfully performing these movements relies on the visuomotor transformation, the translation of the object’s intrinsic properties (e.g., size) into motor or postural commands for the hand and the fingers [8,9,10,11].

Budisavljevic and colleagues [12,13] explored how associative fronto-parietal structural connections subserving visuomotor transformation, namely the branches of the superior longitudinal fasciculus (SLF) [14,15,16,17,18,19], relate to reaching and reach-to-grasp movement, by combining diffusion imaging tractography with three-dimensional recordings of hand movement kinematics. They demonstrated that microstructural properties of the fronto-parietal networks were associated with differences in terms of planning and execution of reaching and reach-to-grasp movements, with special regard to the temporal unfolding of these movements [12].

**Figure 1:**
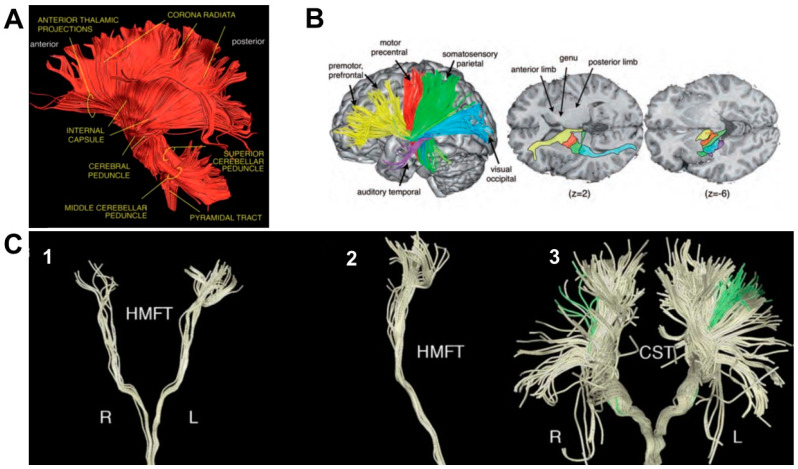
Representative diffusion tensor tractography reconstructions of white matter tracts considered in the study. **Panel A:** left lateral view of the ascending (sensory) and descending (motor) projection pathways forming the corona radiata. **Panel B:** motor and thalamic pathways distribution along the internal capsule. The colored tracts indicate the anterior (yellow), superior (red and green), posterior (blue), and inferior (purple) thalamic peduncles; z values indicate the number of the slice on the z-plane. **Panel C:** hand related motor fiber tracts (HMFTs). (1) front view in the coronal plane and (2) viewed from the right side; (3) front view of the CorticoSpinal Tract (CST–light yellow) and the HMFTs (green) as a subset of CST fibers. R = right; L = left. **Panels A** and **B**: adapted from Catani, M. 2012; **Panel C**: adapted from Dalamagkas, K.; 2020.

Less is known regarding how projection pathways’ microstructure can relate to motor performance. Projection pathways are a complex organization of ascending and descending axons interconnecting ventral and dorsal regions of the central nervous system and conveying sensory and motor information. A crucial node of this system is the internal capsule (IC), a compacted bundle of white matter confined between the putamen and the thalamus, medially to the caudate nucleus [20,21] (Figure 1B), collecting fibers of the corona radiata (CR–Figure 1A), a crown-like shaped structure formed by ascending thalamic fibers radiating out to different cortical areas and descending fibers directed to the base of the cerebrum.

By means of diffusion, MRI tractography IC fibers have been virtually reconstructed, together with fibers originating from the major cortical areas of the motor system and projecting to the cerebral peduncle, highlighting bundles arising from the primary motor cortex (M1), PMv, PMd, supplementary motor area (SMA) and parietal cortex, and traveling ventrally through the CR (Figure 1A) [16,22]). While converging in the IC, the topographic distribution of each set of fibers is maintained, meaning that axons are arranged from front to back according to their origin or destination (Figure 1B) and, in the case of the somatosensory and motor fibers, the parts of the body they serve [20,22].

In the posterior sector of the IC, the corticospinal tract (CST–Figure 1C, panel 3) can be identified, one of the most important descending tracts of the CNS [23,24,25]. The CST originates in the sensorimotor cortex, travels dorsoventrally through the CR, the cerebral peduncles, pons and the medulla and synapse onto the lower motor neurons of the spinal cord [23]. The majority of corticospinal axons forming the CST crosses the midline at the pyramidal decussation and form the lateral CST; the remaining axons travel ipsilaterally and form the anterior CST [26]. The hand motor tract (HMT–Figure 1C, panels 1–2), comprising the hand fibers originating from the primary motor cortex [21,27,28], is of particular interest when considering grasping and reach-to-grasp movements.

How the microstructure of the IC, CST and HMT can relate to motor performance is still quite unexplored: previous studies mainly focus on neurological populations [29,30] and converge in reporting white matter damage as the cause of functional abnormalities. Very recent evidence combining diffusion techniques and non-invasive brain stimulation has highlighted how microstructural differences of the CST (e.g., higher number of streamlines, increased fractional anisotropy, FA) are associated with increased corticospinal excitability and conductivity [4].

Capitalizing on these pieces of evidence, here we focus on how anatomy of hand-related descending projection pathways relates to the temporal kinematics of hand movements, namely reaching and reach-to-grasp. Exploring whether and how the temporal unfolding of hand actions and projection pathways microstructure relate should provide new insights into the comprehension of the neural correlates of motor performance, to be potentially exploited also in the neuro-rehabilitative domain.

## 2. Materials and Methods

Diffusion magnetic resonance imaging tractography relying on the spherical deconvolution approach [31] is adopted to dissect the three considered white matter tracts (IC, CST and HMT) in a healthy adult population. As a measure of white matter microstructure, Hindrance Modulated Orientational Anisotropy (HMOA) [32,33] was considered. HMOA has the potential to estimate water diffusion along each orientation represented within each single voxel of the entire brain volume and can be highly sensitive to modification in fibers diffusivity and organization in terms of microstructural properties. This peculiarity becomes of particular importance when considering white matter regions with complex organization, such as the projection pathways. HMOA data is combined with three-dimensional kinematical measures describing the temporal unfolding of reaching and reach-to-grasp movements, such as peak velocity, peak acceleration and peak deceleration. Overall, these parameters are considered as the typical kinematical fingerprint of distinct phases for both movements [34,35].

### 2.1. Participants

A sample of 31 healthy participants balanced for sex (14 males, 17 females) and equally distributed for age (mean age 24.6 ± 2.70, age range: 20–31 years) was recruited through on-line and local advertisements within the Campus od Padova University. All participants were right-handed according to the Edinburgh Handedness Inventory [36], which ranges from −100 for purely left-handed to +100 for purely right-handed participants. Recruited participants did not report any history of neurological and psychiatric disorders. Informed consent was obtained from all subjects involved in the study, according to the ethics approval as defined by the Institutional Review Board of the University of Padova (Declaration of Helsinki, Sixth revision, 2008).

### 2.2. Kinematics Measurements

#### 2.2.1. Setting and Procedure

**Figure 2:**
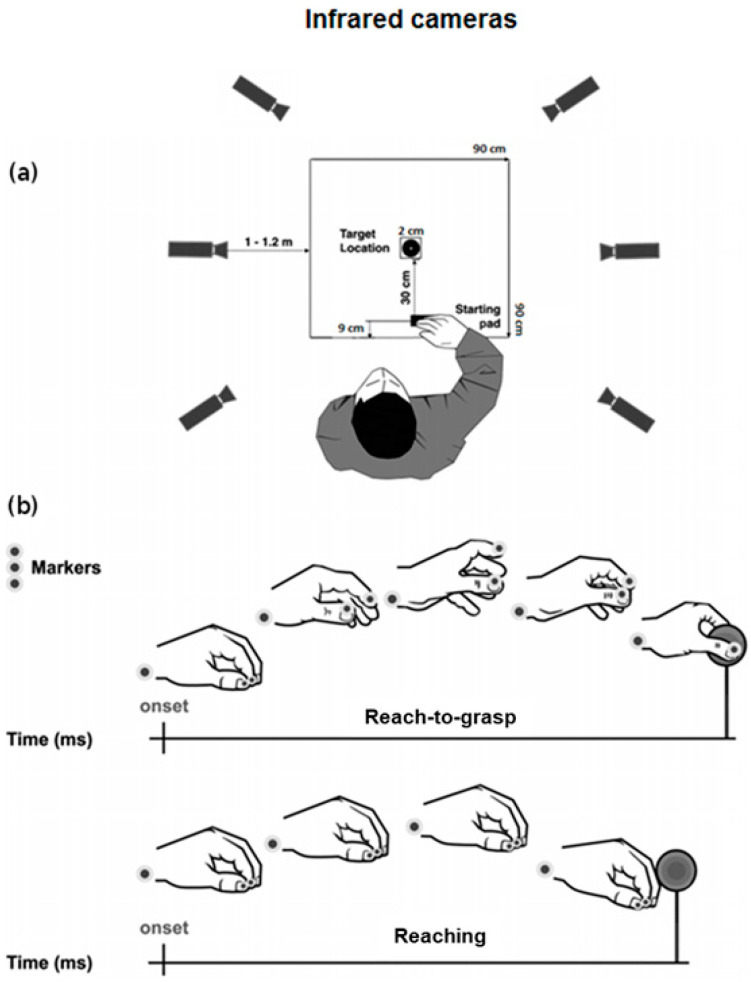
Schematic representation for (**a**) the experimental set-up and (**b**) the reach-to-grasp and the reaching movements together with markers positioning. Adapted from [12].

The experimental setting is represented in Figure 2a. Participants were tested individually in a well-lit room and were seated on a height adjustable chair so that the thorax pressed gently against the front edge of the table (900 × 900 mm) and the feet were supported. Right hand’s position was pronated with the palm resting on a pad (60 × 70 mm), whose shape allowed for a comfortable and repeatable posture of all digits. The starting pad was attached 90 mm away from the edge of the table surface. The target object, consisting of a spherical object (2 cm Ø), was placed 30 cm away from the starting pad, along the mid-sagittal plane of the participant. Participants were requested to perform 2 different tasks: (1) reach-to-grasp, in which they were asked to reach toward and grasp the object by using only index finger and thumb (Precision Grip, PG–Figure 2b); (2) reaching task, in which they were asked to reach the stimulus and touch its frontal surface with their knuckles, maintaining the hand in a closed fist. Participants were instructed to fixate on the target object during both the reaching and the reach-to-grasp actions. Two different auditory cues delivered through headphones informed participants as to which task to perform (high-pitch: reach-to-grasp; low-pitch: reaching). The sound also had the function of telling participants to start their actions toward the object (they were not supposed to start the movement before the auditory cue was presented). Trials in which the participants did not fulfill tasks instructions (e.g., did not fixate the target) were not excluded from the analysis. The two experimental tasks (reaching and reach-to-grasp task) consisted of 30 trials each. The order of the tasks was counterbalanced across participants. The experimental session was preceded by 10 practice trials. The experiment lasted approximately 15 min.

#### 2.2.2. Data Recording

To track the kinematics of the participants’ right hand, we used the 3D-optoelectronic SMART-D system (Bioengineering Technology & Systems, B|T|S|). Three reflective markers (0.25 mm Ø) were taped to the following points on participants’ right upper limbs (see Figure 2b): (1) wrist (radial aspect of the distal styloid process of the radius), (2) thumb (ulnar side of the nail) and (3) index finger (radial side of the nail). The index finger and the thumb markers were used to measure the grasping component of the movement, whereas the wrist marker was used to measure the reaching component. To record the movement, six infrared video cameras (sampling rate 140 Hz) detecting the reflective markers were placed in a semicircle at a distance of 1–1.2 m from the table (Figure 2a). To optimize accurate tracking of all markers, the system was calibrated before data collection by adjusting the camera position, roll angle, zoom, focus, threshold and brightness. These procedures were followed by static and dynamic calibration. For the static calibration, a three-axes frame of 5 markers at known distances from each other was placed in the middle of the table. For the dynamic calibration, a three markers wand was moved throughout the workspace of interest for 60 s. The spatial resolution of the recording system was 0.3 mm over the field of view. The standard deviation of the reconstruction error was 0.2 mm for the x, y and z axes.

#### 2.2.3. Data Processing and Analysis

Each trial was individually checked for correct marker identification and the SMART-D Tracker software package (B|T|S|) was used to provide a 3-D reconstruction of the marker positions as a function of time. The data were then filtered using a finite impulse response linear filter (transition band = 1 Hz, sharpening variable = 2, cut-off frequency = 10 Hz) [37,38]. Movement onset was calculated as the time at which the tangential velocity of the wrist marker crossed a threshold (5 mm/s) and remained above it for longer than 500 ms. For the reach-to-grasp task, the end of movement was defined as the time at which the hand made contact with the target and quantified as the time at which the hand opening velocity crossed a threshold (5 mm/s) after reaching its minimum value and remained under it for longer than 500 ms. For the reaching task, the end of movement was defined as the time at which the hand made contact with the object and quantified as the time at which the wrist velocity crossed a threshold (5 mm/s) after reaching its minimum value and remained above it for longer than 500 ms. For both tasks we considered the kinematic parameters that are commonly used to characterize the considered movements e.g., [39]: movement time (the time from the onset to the end of the movement), the time of maximum peak wrist acceleration (the time at which the maximum wrist acceleration occurred), the time of maximum peak wrist deceleration (the time at which the maximum wrist deceleration occurred) and the time of maximum peak velocity (the time at which maximum peak velocity occurred). For the reach-to-grasp task, we considered three additional grasp-specific measures, namely: the time of maximum grip aperture (the point in time at which the index finger and the thumb reached the maximum distance), the time of the maximum opening velocity (the time at which the tangential velocity of the thumb and index finger reached its maximum value during the opening phase), and the time of the maximum closing velocity (the time at which the tangential velocity of the thumb and index finger was maximum during the closing phase).

### 2.3. Diffusion MRI Measurements

#### 2.3.1. Data Acquisition

Diffusion Imaging Data collection took place after kinematics measurement. Data were acquired by means of a Siemens Avanto 1.5 T scanner, housed in the Radiology Department of Padova University Hospital, with actively shielded magnetic field gradients (maximum amplitude 45 mT/m). An 8-channels head coil was used for RF transmission signal reception. Protocol included a localizer scan, followed by a single-shot, spin-echo, EPI sequence (TR = 8500, TE = 97, FOV = 307.2 × 307.2, matrix size = 128 × 128, 60 slices without gaps, with isotropic voxel (2.4 × 2.4 × 2.4 mm^3^). The maximum diffusion weighting was 2000 s/mm^2^, 7 images were acquired at each slice location (no diffusion gradients applied; b = 0 s/mm^2^), together with 64 diffusion-weighted images (gradient directions were uniformly distributed in space and repeated 3 times, to increase signal to noise ratio). Gains and scaling factors were kept constant between acquisitions. Scanning lasted approximately 30 min.

#### 2.3.2. Head Motion and Eddy Current Distortions Correction and Estimation Distribution of Fibers Orientation

Before analysis, raw images were examined to detect outliers, including signal dropouts, poor signal-to-noise ratio and image artifacts such as ghosts. Any subject whose raw data contained significant image quality issues was discarded from further analyses. The remaining 31 participants were processed as follows: the 4 repeated Diffusion Weighted Imaging datasets were concatenated and corrected for motion and geometrical distortions using ExploreDTI (http://www.exploredti.com accessed on 1 August 2022) [40]. Spherical deconvolution [41,42] approach was chosen to estimate multiple orientations in voxels containing different populations of crossing fibers [43]. Spherical deconvolution was calculated applying the damped version of the Richardson–Lucy algorithm with a fiber response parameter α = 1.5, 200 algorithm iterations, and η = 0.15 and ν = 15 as threshold and geometrical regularization parameters [31]. Fiber orientation estimates were obtained by selecting the orientation corresponding to the peaks (local maxima) of the fiber orientation distribution (FOD) profiles. To exclude spurious local maxima, both an absolute and a relative threshold on the FOD amplitude were applied [32]. A first “absolute” threshold, corresponding to Harvard-Oxford Atlas (HOA) threshold of 0.015, was chosen to exclude intrinsically small local maxima due to noise or partial voluming effects with isotropic tissue. This threshold was set to select only the major components for fiber orientation and to exclude spurious low amplitude components of fiber orientation (Fiber Orientation Distribution, FOD) obtained from gray matter and cerebro-spinal fluid isotropic voxels. A second “relative” threshold of 5% of FOD maximum amplitude was applied to exclude remaining unreliable local maxima with values greater than the absolute threshold but still significantly smaller than the main fiber orientation [32].

#### 2.3.3. Tractography Algorithm

Whole brain tractography was performed, selecting every brain voxel with at least 1 fiber orientation as a seed voxel. From these voxels, and for each fiber orientation, streamlines were propagated using a modified Euler integration with a step size of 1 mm. When entering a region with crossing white matter bundles, the algorithm followed the orientation vector of the least curvature. Streamlines were halted when a voxel without fiber orientation was reached or when the curvature between 2 steps exceeded a threshold of 45°. All spherical deconvolution and tractography processing was performed using StarTrack, a freely available Matlab software toolbox developed by Flavio Dell’Acqua at NatBrainLab, King’s College London, and based on the methods described in Dell’Acqua et al. [32].

#### 2.3.4. Tractography Dissections of the Internal Capsule, Corticospinal Tract and Hand Motor Tract

The TrackVis software was used to visualize fiber tracts and quantify tract-specific measures (http://www.trackvis.org accessed on 1 August 2022) [44].

**Figure 3:**
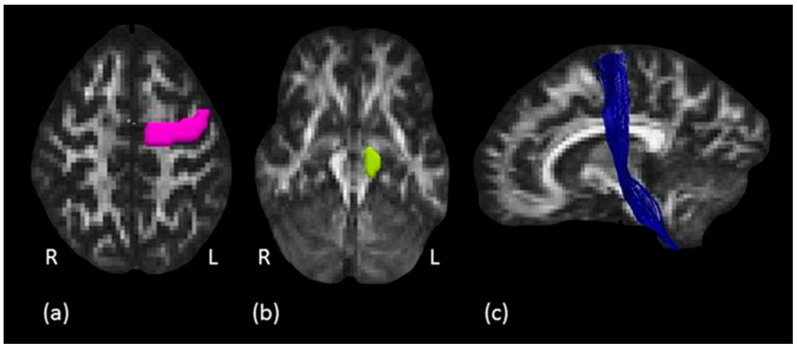
Corticospinal tract tractography dissection. (**a**) The pink ROI is defined around the left precentral gyrus on axial plane and (**b**) the green ROI defined around the left cerebral peduncle. (**c**) In blue, example of the left corticospinal tract in a representative subject, sagittal view.

Internal capsule: a single region of interest (ROI) approach was used to dissect the internal capsule (IC) as described by Catani and Thiebaut de Schotten [21]. The ROI was defined around the anterior and posterior arms of the IC on several consecutive axial slices on the Fractional Anisotropy (FA) map of each subject. The upper limit of the ROI was defined by the slice where the lenticular nucleus separates the internal from the external capsule. The ROI was drawn on each slice until the point where the anterior commissure comes visible separating the anterior and posterior arms of the IC. A “NOT” ROI was used to exclude the undesired fibers of the corpus callosum projecting to the contralateral hemisphere. Using another “NOT” ROI all the streamlines of the cingulum projecting to the temporal lobe were excluded. An example of tractography reconstruction in a representative subject, together with the IC ROI used, is shown in Figure 3.

Corticospinal tract: to isolate the CST, a two ROIs approach was used as described by Rojkova et al. [45]. The first ROI was drawn around the precentral gyrus of the primary motor cortex (M1) on several consecutive axial slices where the central sulcus (CS) was clearly identifiable. The second ROI was defined around the cerebral peduncle (CP) to create an obligatory passage for the fibers corresponding to the pyramidal tract, originating from the precentral gyrus and traveling down the brainstem to the spinal cord (Figure 3). A “NOT” ROI was used to exclude undesired fibers passing through the ROIs but not projecting to the motor cortex. An example of tractography reconstruction in a representative subject, together with the two ROIs used, is shown in Figure 3.

**Figure 4:**
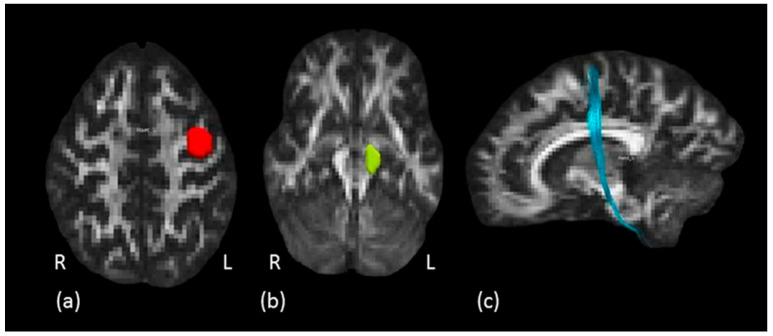
Hand motor tract dissection. (**a**) The red ROI is defined around the left knob-like structure on axial plane and (**b**) the green ROI is defined around the left cerebral peduncle. (**c**) In light blue, example of left hand motor fibers tractography reconstruction in a representative subject, sagittal view.

Hand motor tract: to isolate the HMT, i.e., the hand fibers of the pyramidal CST tract, a two ROIs approach was used. First, the anatomical hand motor cortex (HMC) was localized on the axial plane at the level of the segment of the precentral gyrus with an omega-like shape [46]. This ROI was defined on the knob-like structure of the omega sign described above, on several consecutive axial slices. The second ROI was drawn around the cerebral peduncle to create an obligatory passage for the HMT fibers, originating from the hand area of precentral gyrus (first ROI) and traveling down the brain stem to the spinal cord. A “NOT” ROI was used to exclude undesired fibers passing through the ROIs but not projecting to the hand motor area. Two subjects were excluded from the analysis because it was not possible to reconstruct the HMT. An example of tractography reconstruction together with the ROIs as described above is shown in Figure 4.

#### 2.3.5. Data Processing and Analysis

All statistical analyses were performed using the computing environment R (R Core Team) and adopting a Bayesian approach [47]. Briefly, in Bayesian estimation, the aim is to allocate credibility to a distribution of possible parameter values (posterior distribution) consistent with the observed data, by generating a large number of samples by using a Markov Chain Monte Carlo method (MCMC). Next, we calculated 99% credible intervals (99% CI) to determine whether the differences between conditions were credibly different from zero. We considered a difference plausible if the 99% CI excluded zero. A total of 10,000 iterations was used to improve the precision of estimations. All statistical analyses were performed.

## 3. Results

### 3.1. Kinematics Data

Statistically significant differences among the two types of actions for the kinematic parameters (see Table 1) were observed [48]. A principal component analysis (PCA) with Oblimin rotation was applied to decompose the data into their underlying factors and reduce the number of kinematic variables so as to obtain a protection against the Type I error.

A Principal Component Analysis (PCA) with Oblimin rotation for each task was performed. On the basis of Kaiser’s rule [49], we selected components having eigenvalues above 1. For the reaching task, we selected one component, namely a global descriptor of the reaching timing (Table 2), comprising the total movement duration, the time of maximum peak velocity, the time of maximum peak wrist acceleration and deceleration. The selected component accounted for 81% of the variance. Similarly, for the reach-to-grasp task we selected one component, namely a global descriptor of the reach-to-grasp timing (Table 2), including the total movement duration, the time of maximum peak velocity, the time of maximum peak wrist acceleration and deceleration, the time of maximum grip aperture and the time of maximum opening velocity. The selected component accounted for 86% of the variance.

### 3.2. Diffusion Imaging Data

For each tract, namely the IC, the CST and the HMT, the HMOA was calculated. We limited our structural investigation to HMOA because this metric has shown ability to (1) resolve partial volume contamination of different white matter tracts crossing within the same voxel or brain region and (2) provide a distinct and therefore a more “true” tract-specific quantification of anisotropy and microstructural organization along each white matter tract (i.e., if two fibers are crossing the same voxel, two distinct and independent HMOA values are assigned to each tract) [32]. Lateralization index (LI) for IC, CST tracts and HMT was computed (Table 3) for HMOA using the following formula described by [16]:

LIHMOA = [(right HMOA) − (left HMOA)/[(right HMOA) + (left HMOA)](1)

The value of the LI reflects the lateralization of the tract: positive values are suggestive of a rightward asymmetry, whereas negative values indicate leftward asymmetry. Values around zero indicate symmetrical organization between the left and right segments. Using the R’s package BEST (Bayesian ESTimation supersedes the t test), we performed a One-sample Bayesian t-test to assess whether lateralization was credibly different from zero.

Finally, to detect the strength of the correlation between HMOA and the kinematical components (Reaching timing and the Reach-to-Grasp timing) we performed a Bayesian correlational analysis using R’s package BayesianFirstAid. The Bayesian correlations between HMOA and the Reaching and the Reach-to-Grasp timing components are shown in Table 4.

#### 3.2.1. Reaching Task

For all the considered projection pathways (IC, CST and HMT), the reaching timing component positively correlated with HMOA in the left but not in the right tract (Figure 4, panel a).

#### 3.2.2. Reach-to-Grasp Task

**Figure 5:**
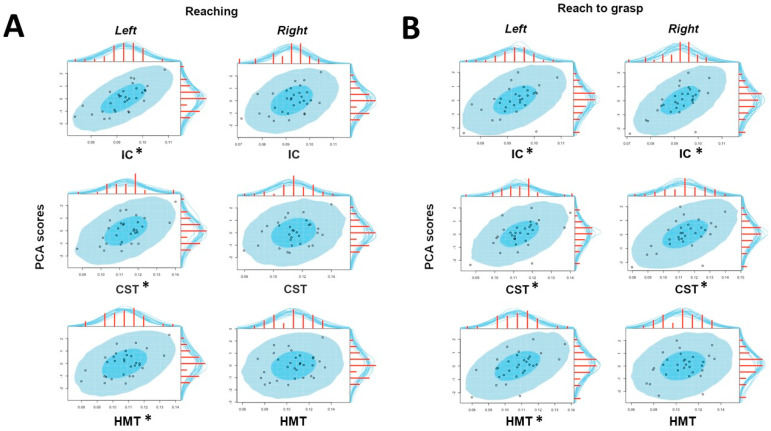
Graphical representation of the Bayesian correlations between HMOA values (x-axis) and PCA components (y-axis) obtained for both tasks (**Panel A**: reaching; **Panel B**: reach-to-grasp) for the considered tracts (IC, CST, HMT) in both hemispheres (left, right). *: significant correlation.

The Reach-to-grasp timing component was credibly and bilaterally correlated with the HMOA of the IC and the CST. Regarding the HMT, the correlation between the Reach-to-grasp timing component and the HMOA was credibly different from zero only for the left pathway (Figure 4, panel b). Results are graphically summarized in Figure 5.

## 4. Discussion

In the present study, white matter pathways of the internal capsule (IC), the corticospinal tract (CST) and the hand motor tract (HMT) have been reconstructed using state-of the-art spherical deconvolution tractography (HMOA). HMOA results, together with PCA components of kinematics parameters describing the temporal profile of reaching and reach-to-grasp movements, have been analyzed within the framework of a Bayesian correlation. The results show how HMOA for these tracts relate with the kinematics of reach-to-grasp and reaching only movements in a healthy right-handed population. Overall, this results converge with the current literature in showing that reaching and reach-to-grasp movements are characterized by a different profile in terms of temporal kinematics [35,39,48]. This is an important aspect upon which build the discussion concerned with the role played by the considered neural structures in shaping the timing of kinematical parameterization.

### 4.1. White Matter Asymmetries

In terms of lateralization, HMOA results point towards an absence of significant asymmetry in terms of white matter microstructure in the IC, the CST and the HMT. These findings are partially in agreement with previous literature, which mainly adopts microstructural measures such as FA or MD, and only in a few cases relies on other measures such as anterior–posterior direction (ADCap). However, the findings are rather controversial: regarding CST lateralization, only a few studies reported symmetrical distribution [16,50], while others observed leftward [51,52,53,54] or rightward [55] lateralization.

Concerning the IC, leftward asymmetry for the posterior branch has been observed by using a voxel-based morphometry (VBM) [56] and diffusion [54,57,58,59] analyses. The HMT has been explored less, with one study highlighting the same leftward asymmetry profile as the CST, though with higher levels of inter-subject variability [60]. A point worth noting is that the above mentioned studies rely on diffusion measures such as Fractional Anisotropy (FA) or Mean Diffusivity (MD): here we used the HMOA index, an SD-based measure which is much less affected by fibers orientation and thus more reliable than the FA [32]. The FA index is known being strongly affected by the presence of fibers with different orientations in the same voxel [31] and this might explain the discrepancy among previous results concerning the FA lateralization of the IC and CST. Indeed, the IC, the CST and the HMT are located in brain regions characterized by a complex white matter architecture, where projection fibers are crossed by associative (i.e., superior longitudinal fasciculus) and commissural pathways (i.e., transcallosal fibers) leading to a limited possibility of properly identifying anisotropy conditions and therefore bringing misleading results if explored by means of FA [50,61].

Studies exploring white matter projection pathways usually do not report asymmetries in diffusion parameters according to laterality [62]: the results described here converge with previous evidence. However, if laterality does not seem to play a role in pure anatomical terms, it does when HMOA is put into correlation with kinematic parameters describing temporal aspects of motor behavior: HMOA of left projection pathways—supporting actions performed with the right hand—appear to be coherent with the movement timing for both reaching and reach-to-grasp. Yet, when considering grasping, the positive correlation between HMOA and temporal kinematics extends to the right IC and CST, but not to the HMT. Overall, these results seem to point towards a possible link between the organization of projection pathways mediating hand movements and motor dominance, a peculiarity of human motor control, as already suggested by a study combining diffusion measures and task complexity and showing a bilateral and widespread recruitment of neural tracts as a function of task complexity [63]. In fact, although anatomically symmetrical, our hands are often used according to a quite asymmetrical pattern: the vast majority of human beings shows a preference for using one hand for dealing with a great variety of tasks, while the other hand plays a more supporting role, especially in the context of bimanual actions. For about 90–95% of cases the right hand acts as the dominant [64,65] while in only about 5–10% of cases is this pattern reversed [66]. Our participants are classified as right-handed [36] and according to the literature left projection pathways—supporting actions performed with the right hand—should appear as more represented. In this respect, transcranial magnetic stimulation (TMS) and brain imaging evidence are not consistent: some studies observe unilateral and contralateral recruitment of the visuomotor circuit during unimanual grasping task performed with the right hand [67,68,69], while others describe a bilateral activation pattern [70,71,72]. From this perspective, the result of a bilateral positive association between kinematics and HMOA parameters is not totally surprising and provides further support to the second hypothesis. Regarding HMT, the final segment of the CST specifically devoted to convey information to the distal muscles of hand fingers, the positive association between HMOA and grasp timing is limited to the left tract. Previous studies report a clear association between diffusion parameters of projection pathways and temporal aspects of task execution, such as reaction time [73,74,75], leading to the hypothesis that temporal aspects of action execution might be more specifically mirrored by ventral regions of bilateral descending pathways (such as the CST and the IC), rather than more dorsal ones, such as the HMT. Overall, this result points toward a possible link between white matter organization of projection pathways and motor performance related to the temporal organization of unfolding actions.

### 4.2. Relating Variation in Kinematics to the Internal Capsule

The IC is a complex bundle of white matter fibers containing both ascending afferent and descending efferent fibers which connect the core sensorimotor cortical areas, namely the primary motor cortex (M1), the dorsal premotor cortex (PMd), the ventral premotor cortex (PMv), the posterior parietal cortex (PPC), and the occipital cortex with the lower part of the cerebrum and the spinal cord [15,17]. The important interplay between fronto-parietal areas in shaping, implementing and monitoring motor programs has been the subject of much investigation [1,9,76,77]. The findings of the present study provide insights on how projecting fibers connecting the fronto-parieto-occipital cortex and the spinal cord relate to kinematic movement parameters reflecting visuomotor processing. Results showed that the HMOA of the bilateral IC positively correlates with the timing component of the reach-to-grasp movements (but not with the same aspect of reaching movements, where this association is significant only for the left tract). This suggests a specific involvement of the bilateral IC in the programming of prehensile actions, which characterizes grasping, but not reaching movements. Specifically, this might suggest a more accurate specification of the finger movement trajectories and greater control of the hand dynamics required by grasping movement [78]. Finger extension constitutes the initial fundamental phase of the grasping movement [39,79] and it seems to rely on the recruitment of extensor muscles [80]. In stroke patients, the inability to properly extend the digits affects the hand shaping and therefore the possibility of performing an adequate grasping [81]. Furthermore, this ability represents a reliable predictive measure of motor recovery outcome [82]. In the current study, the results suggest that the white matter microstructural organization of the bilateral IC is involved in the control of the grip component. This possibly supports an efficient visuomotor transformation used to shape the motor program and to activate the hand’s muscles to an appropriate extent. This idea is in line with previous lesion studies reporting dysfunctional movements in patients affected by capsular stroke compared to controls [83]. Overall, it seems that the IC macrostructure could play a role in determining how kinematic markers specifically related to grasping are temporally parameterized [84,85].

### 4.3. Relating Variation in Kinematics to the Corticospinal Tract

The crucial role of the CST and its cortical origin—the primary motor cortex (M1) in movement control—is well established [24]. Evidence comes from functional magnetic resonance imaging (fMRI) studies showing activity of the M1 during both reaching and grasping movements [86,87,88,89,90]. Furthermore, lesion studies [91,92] together with findings of brain stimulation studies [93], added important causal evidence of the importance of the pyramidal fibers’ integrity in performing fine discrete movements of the distal musculature. Recently, diffusion MRI tractography allowed investigation of white matter changes in the CST because of a range of diseases including amyotrophic lateral sclerosis (ALS) [55,94], multiple sclerosis [95,96] and acute lesions [97]. In stroke patients, reduced FA has been observed in the ipsi-lesional CST compared to the unaffected hemisphere and compared to the control group [98]. Furthermore, the reduction of FA within the CST, interpreted as white matter disintegration, was associated with the severity of the motor deficits following stroke lesion [99] and with the possibility of recovery [97].

To date, little attention has been given into how the anatomy of the CST, in a healthy population, influences and mediates different kinematic parameters of hand movements: the present study is the first to provide insights into how the microstructural organization of the CST is correlated with the kinematical timing of reach-to-grasp movements. Our results are in line with the evidence that in humans and monkeys CST fibers are essential for fingers control [100,101,102], and reflect a contribution of the CST to the on-line control of the hand while approaching to the target object [103]. Overall, our findings point towards a bilateral involvement of the CST during reach-to-grasp movements and the involvement of the left CST only when it comes to reaching. Even though a wealth of evidence reported only contralateral activity in M1 during reach-to-grasp [9,90,91], bilateral functional involvement of M1 has been previously observed during unilateral hand movements in healthy subjects [104,105]. Recruitment of the ipsilateral M1 becomes more evident during motor recovery after brain lesion [89]: in stroke patients reporting unilateral brain damage involving precentral cortex bilateral movement abnormalities have been observed. Yarosh et al. [106] tested patients with pyramidal stroke, finding that not only were they profoundly impaired in the contra-lesional limb, but also movements of the ipsi-lesional hand lacked speed and smoothness compared to healthy controls. These findings, together with the results of the present study, suggest a role of CST of both hemispheres in controlling unilateral prehensile actions.

### 4.4. Relating Variation in Kinematics to the Hand Motor Tract

To date, only few studies have tried to localize the HMT; however, there is no consensus on its cortical topographical position. By means of fMRI, some studies have identified a spot-like region of activity in the pre-central gyrus, known as the hand-knob area [107], while others reported hand fibers arising also from other regions of the pre- and post-central gyrus [108]. Furthermore, lesion studies claim the possibility that finger movements are not controlled by a spatially and functionally discrete group of neurons but rather depend on several brain regions and structures [109]. In the present work, we isolated the HMT selecting only the fibers arising from the omega-like structure [31,46]. The results showed an involvement of the hand fibers in both reaching and reach-to-grasp conditions, but the significant correlation between kinematics parameters and HMOA was observed only for the left HMT. This might reflect an involvement of the HMT in modulating and nesting at the appropriate time kinematic parameterization for a successful contact with the target object [34,103,110]. This finding is in line with evidence pointing towards a specific involvement of the left hemisphere in controlling a steady and accurate contact with the object [111] and is consistent with the literature reporting that pyramidal fibers are essential for finger control [100,101,102].

### 4.5. A Note on Limitations of the Study

There are technical limitations that need to be acknowledged in relation to the findings of the present study. White matter pathways architecture can assume sophisticated configurations, such as fibers with different orientation crossing each other (crossing fibers), partially overlapping v-shaped bundles with opposite orientation (kissing fibers) or fibers diverging with a fan-like shape (fanning fibers). All these conditions can influence the accuracy of pathways reconstruction, and may lead to erroneous diffusion estimates. For this reason, spherical deconvolution tractography has been adopted, with the aim of providing a better estimate of fiber orientation in regions where two or more tracts cross at the same point [31]. The use of this technique is especially well grounded when we consider that the number of voxels containing two or more fiber populations in the brain is estimated to be around 90% [112]. Nevertheless, tractography reconstructions based on the spherical deconvolution are still likely to generate false positives [113]. For this reason, all the considered tracts were visually inspected to confirm their known anatomical trajectories.

## 5. Conclusions

This study is the first to demonstrate that hand kinematics and visuomotor processing underlying grasping is associated with the microstructure of the projection pathways, namely IC, CST and HMT. Overall, the results point towards a possible involvement of the projection system in the temporal coupling of the reaching and grasping kinematic markers, in the specification of the temporal aspects concerned with the unfolding of movement trajectory and in guiding a stable contact with the target object. Despite the presence of possible sources of uncertainties that must be considered, the present work puts forward theoretical and methodological implications that bring important insights to the field of neural control of movement. Our observation of consistent convergence between hand motor behaviors and structural connectivity increases our understanding of structure–function relationships. Improving models of motor system developmental plasticity has the potential to provide useful insights for the study of peculiar aspects of motor control such as coordination, in healthy and pathological conditions [114,115], helping in the establishment of imaging predictors of responsiveness to therapeutic interventions. Such efforts may help to improve ad-hoc rehabilitation trainings and long-term outcomes for neurological disorders.

## Figures and Tables

**Table 1:** Mean, standard deviation and Confidence Intervals (CI) for the considered kinematic measures. Statistics comparing the two types of movement are also included.

	Mean		Standard Deviation		99% CI
Variable	Reaching	Reach-to-Grasp	Reaching	Reach-to-Grasp	
Movement Time (ms)	655.13	730.46	55.05	55.81	−119, −41
Time of Maximum Peak Velocity (ms)	263.90	308.76	23.52	24.60	−61, −27
Time of Maximum Peak Acceleration (ms)	165.13	218.83	15.39	12.58	−98, −62
Time of Maximum Peak Deceleration (ms)	424.50	503.73	29.92	20.91	−64, −44
Time of Maximum Grip Aperture (ms)	N/A	509.26	N/A	22.20	N/A
Time of Maximum Opening Velocity (ms)	N/A	289.45	N/A	30.02	N/A

**Table 2:** Weights of the kinematic parameters for the components of the reaching and the reach-to-grasp task.

	Reaching Timing	Reach-to-Grasp Timing
Movement Time (ms)	0.83	0.73
Time of Maximum Peak Velocity (ms)	0.59	0.76
Time of Maximum Peak Acceleration (ms)	0.66	0.59
Time of Maximum Peak Deceleration (ms)	0.91	0.84
Time of Maximum Grip Aperture (ms)	N/A	0.83
Time of Maximum Opening Velocity (ms)	N/A	0.77

**Table 3:** Lateralization Index of IC, CST and HMT calculated on the basis of HMOA values.

LIHMOA	Mean	Standard Deviation	99% CI
IC	0.00	0.01	−0.01, 0.01
CST	0.00	0.04	−0.02, 0.02
HMT	0.06	0.06	−0.02, 0.04

**Table 4:** Bayesian correlations between HMOA and the Reaching and the Reach-to-Grasp timing components. HDI: High Density Interval. Credible interval in parentheses; * indicate credible correlations.

		Reaching Timing [99% CI]	Reach-to-Grasp Timing [99% CI]
IC	Left	*r* = 0.55[0.35, 0.89] *	*r* = 0.52[0.11, 0.81] *
	Right	*r* = 0.34[−0.12, 0.70]	*r* = 0.58[0.12, 0.79] *
CST	Left	*r* = 0.52[0.08, 0.80] *	*r* = 0.50[0.09, 0.81] *
	Right	*r* = 0.32[−0.22, 0.66]	*r* = 0.58[0.09, 0.78] *
HMT	Left	*r* = 0.52[0.07, 0.77] *	*r* = 0.57[0.08, 0.77] *
	Right	*r* = 0.34[−0.62, 0.60]	*r* = 0.28[−0.11, 0.75]

## Data Availability

The data presented in this study are available on request from the corresponding author. The data are not publicly available due to data ownership regulations and privacy regulations contained in the informed consent signed by participants involved in the study.
